# Systematic review and meta-analysis on the adverse events of rimonabant treatment: Considerations for its potential use in hepatology

**DOI:** 10.1186/1471-230X-9-75

**Published:** 2009-10-09

**Authors:** Norberto C Chavez-Tapia, Felix I Tellez-Avila, Giorgio Bedogni, Lory S Crocè, Flora Masutti, Claudio Tiribelli

**Affiliations:** 1 Centro Studi Fegato (CSF) - Liver Research Center, Bldg Q - AREA Science Park-Basovizza Campus, Italy; 2 Department of Gastroenterology Instituto Nacional de Ciencias Médicas y Nutrición Salvador Zubirán, México; 3 Department ACADEM, University of Trieste, Italy

## Abstract

**Background:**

The cannabinoid-1 receptor blockers have been proposed in the management of obesity and obesity-related liver diseases (fatty liver as NAFLD or NASH). Due to increasing number of patients to be potentially treated and the need to assess the advantage of this treatment in terms of risk/benefit, we analyze the side events reported during the treatment with rimonabant by a systematic review and meta-analysis of all randomized controlled studies.

**Methods:**

All published randomized controlled trials using rimonabant *versus *placebo in adult subjects were retrieved. Relative risks (RR) with 95% confidence interval for relevant adverse events and number needed to harm was calculated.

**Results:**

Nine trials (n = 9635) were considered. Rimonabant 20 mg was associated with an increased risk of adverse event (RR 1.35; 95%CI 1.17-1.56), increased discontinuation rate (RR 1.79; 95%CI 1.35-2.38), psychiatric (RR 2.35; 95%CI 1.66-3.34), and nervous system adverse events (RR 2.35; 95%CI 1.49-3.70). The number needed to harm for psychiatric adverse events is 30.

**Conclusion:**

Rimonabant is associated with an increased risk of adverse events. Despite of an increasing interest for its use on fatty liver, the security profile and efficacy it is needs to be carefully assessed before its recommendation. At present the use of rimonabant on fatty liver cannot be recommended.

## Background

As the consequence of important biologic and social modifications, in the last century new non-infectious epidemic diseases appeared as the most important causes of mortality in worldwide. Among these new epidemic diseases, obesity gained a leader position since the pathologic accumulation of adipose tissue has important deleterious effect on life expectancy [1,2]. The metabolic syndrome, of which obesity is the most common cause, is considered the most important risk factor for cardiovascular diseases and other chronic diseases [3].

The algorithm for the management of obesity includes non-pharmacologic, pharmacologic and surgery-based strategies [4]. Several drugs are recommended in the pharmacological approach and in the last years the cannabinoid-1 receptor blocker rimonabant has been proposed as a potential effective therapeutic approach in the management of obesity [5].

Non-alcoholic fatty liver disease (NAFLD), the hepatic manifestation of the metabolic syndrome [6,7] has been shown to be present in more that 70% of the obese subjects [8]. There is a consensus that the key mechanism of hepatic steatosis is the insulin resistance [9]. This observation prompted to investigate new drugs which may be useful in reducing the fatty content in the liver, the cornerstone in the treatment of NAFLD [10]. Although effective, dietary modifications and increased physical activity are associated with a low rate of compliance in the real life since recurrence is almost the rule [11,12]. This explains the increased interest shown by both clinicians and industry to find pharmacological approaches. Based on the favorable results of randomized controlled trials assessing rimonabant in the management of obesity, there is an increasing interest to assess the efficacy of this drug in NAFLD. However data of ADAGIO-Lipids trial showed in NAFLD patients a significant improvement of liver function test and liver fat [13], the histological evidence was not evaluated and the two randomized controlled trials on NAFLD [14,15] were stopped prematurely due to request of national health authorities. Small, uncontrolled observations suggested that rimonabant may be an effective strategy in fatty liver [16] in spite of some adverse events [17]. The adverse events have been so far analyzed by meta-analysis of data from Rimonabant In Obesity (RIO) trials [18,19]. In this review we extend the study on the side events reported during the treatment with rimonabant by a systematic review and meta-analysis of all randomized controlled studies using this cannabinoid-1 receptor blocker in any pathological condition.

## Methods

### Criteria for considering studies for this review

We considered all published randomized controlled trials using rimonabant (any dosage) versus placebo for at least 12 weeks in adults for any clinical indication, regardless of the language of publication. The outcomes were the adverse events reported classified as: 1) any adverse event; 2) serious adverse events; 3) discontinuation due to adverse event; 4) discontinuation due psychiatric adverse event; and 5) discontinuation due to neurologic adverse event.

### Search methods for identification of studies

Reported randomized clinical trials were identified by search of The Cochrane Hepato-Biliary Group Controlled Trials Register (May 2009), The Cochrane Library (Issue 2, 2009), EMBASE (January 1980 to May 2009), and MEDLINE (January 1966 to May 2009). Search terms were ("rimonabant" OR "Acomplia" OR ["antagonist" AND "cannabinoid" AND "receptor"]) AND ("randomized controlled trial" OR "random" OR "blinded" OR "controlled") AND "placebo". The references of all identified studies were inspected for more trials. We also checked for any missing trials by examining the references of existing reviews of this topic.

Two authors (NCT, FTA) independently reviewed the search output of potentially relevant trials for inclusion and assessed the trial for inclusion. Studies not meeting the inclusion criteria were excluded. Disagreements were settled by discussion with a third co-author (CT). In an independent manner we assessed the bias risk by the following components of methodological quality of included studies: generation of allocation sequence, allocation concealment, blinding, incomplete outcome data and selective outcome reporting [20].

### Statistical analysis

We use the software package RevMan 5 [21]. For dichotomous variables, we calculate the relative risk (RR) with 95% confidence interval. A fixed effect model was used throughout the review, except in the event of significant heterogeneity between the trials (P < 0.10), when the random effect model was chosen. Heterogeneity was explored by chi-squared test with significance set at P-value 0.10, and the quantity of heterogeneity was measured by I^2 ^[22]. We use a funnel plot to explore bias. Asymmetry in funnel plot of trial size against treatment effect was used to assess this bias. We perform linear regression approach described by Egger *et al*. to determine the funnel plot asymmetry [23]. Number needed to harm was calculated.

## Results

**Figure 1:**
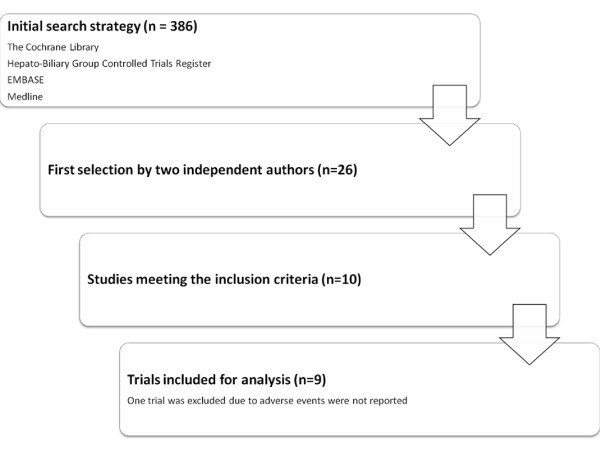
**Flow chart for search strategy and selection of trials**.

The initial search strategy included 386 references. Out of these studies, 26 studies were considered further and 10 randomized controlled trials were finally found suitable for the analysis [13,24-32]. Out of the 10 trials one was excluded because no adverse effects were reported (Figure 1) [31].

The characteristics and quality of studies included are reported in Table 1. All trials had a low risk of bias. Five trials were designed to assess the efficacy on weight reduction, one for the percent change of atheroma volume, one for the management of alcohol dependency, one to assess the changes in HbA_1c_; one for hyperandrogenaemia; one for change in lipid profile, and one was designed to assess insulin resistance in patients with polycystic ovary syndrome. The last one was the excluded study since the adverse events were not reported. The longest follow-up was 24 months with a range between 3 and 24 months. A pooled 2849 subjects were included in the placebo arm, 2883 in rimonabant 5 mg arm, and 3903 were assigned to rimonabant 20 mg. In the serious adverse events, discontinuations for adverse events and psychiatric adverse events analysis biases were observed in the funnel plot graph (Additional file 1).

**Table 1:** Methodological quality summary: review authors' judgments about each methodological quality item for each included study.

Author (year)	Subjects included	Duration (months)	Primary outcome	Adequate sequence generation	Allocation concealment	Blinding	Incomplete outcome data	Selective outcome reporting
Van Gaal (2005)*	placebo = 305	12	Weight change	Yes	Yes	Yes	No	No
	rimonabant 5 mg = 603							
	rimonabant 20 mg = 599							
Després (2005)*	placebo = 342	12	Weight change	Yes	Yes	Yes	No	No
	rimonabant 5 mg = 345							
	rimonabant 20 mg = 346							
Pi-Sunyer (2006)*	placebo = 607	12	Weight change	Yes	Yes	Yes	No	No
	rimonabant 5 mg = 1214							
	rimonabant 20 mg = 1219							
Scheen (2006)*	placebo = 348	12	Weight change	Yes	Yes	Yes	No	No
	rimonabant 5 mg = 358							
	rimonabant 20 mg = 339							
Nissen (2008)	placebo = 417	18	Change in percent atheroma volume	Yes	Yes	Yes	No	No
	rimonabant 20 mg = 422							
Van Gaal (2008)*	placebo = 168	24	Weight change	Yes	Yes	Yes	No	No
	rimonabant 5 mg = 363							
	rimonabant 20 mg = 305							
Soyka (2008)	placebo = 127	3	Time to first drink	Yes	Yes	Yes	No	No
	rimonabant 20 mg = 131		Time to relapse to first heavy drinking					
Rosenstock (2008)	Placebo = 140	6	HbA_1c_	NR	NR	Yes	No	No
	Rimonabant 20 mg = 138							
Després (2009)	Placebo = 395	12	Change in HDL cholesterol and triglycerides	Yes	Yes	Yes	No	No
	Rimonabant 20 mg = 404							

* The articles with asterisk are part of Rimonabant In Obesity (RIO) trials

### Adverse events

**Figure 2:**
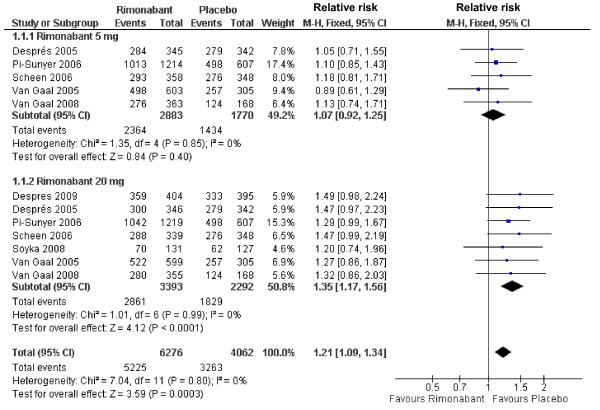
**Forest plot graphic assessing the risk to be free of any adverse events at rimonabant dosages of 5 and 20 mg**.

**Figure 3:**
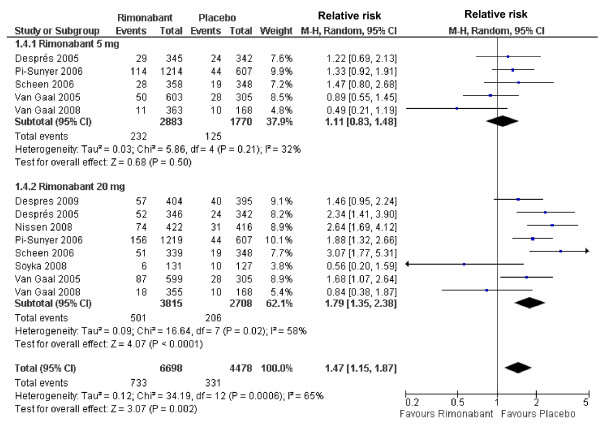
**Forest plot graphic assessing the risk to be free of discontinuation due to adverse events at rimonabant dosages of 5 and 20 mg.**.

The use of rimonabant at dosage of 5 mg was not associated with any adverse event. Conversely, the use of 20 mg was associated with an increased risk for adverse event *vs*. placebo (RR 1.35; 95%CI 1.17-1.56), with a pooled effect of increased risk (RR 1.21; 95%CI 1.09-1.34) (Figure 2). By assessing only serious adverse events, a trend for increased risk in the rimonabant groups was observed at both dosages (5 and 20 mg), although the difference did not reach statistical significance (RR 1.16; 95%CI 0.93-1.45) (Additional file 2). In the subjects receiving 5 mg rimonabant, no difference was found in the discontinuation rate for adverse events while in those receiving 20 mg rimonabant an association between drug use and discontinuation rate (RR 1.79; 95%CI 1.35-2.38) was observed. The same was found when data obtained in pooled series (5 and 20 mg of rimonabant) were considered (RR 1.47; 95%CI 1.15-1.87 for) (Figure 3).

**Figure 4:**
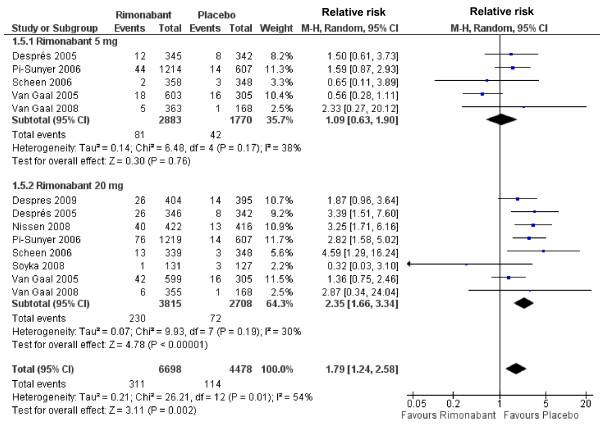
**Forest plot graphic assessing the risk to be free of treatment discontinuation due to psychiatric adverse events at rimonabant dosages of 5 and 20 mg**.

**Figure 5:**
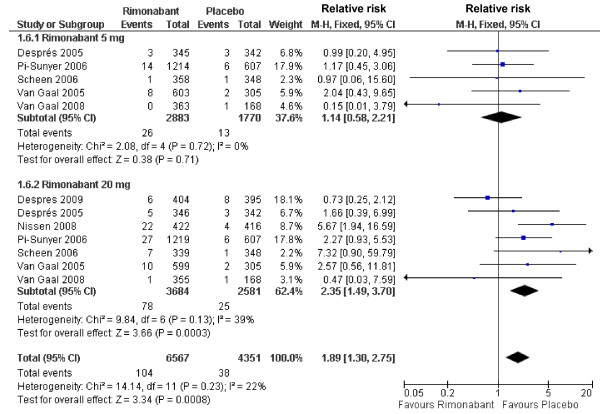
**Forest plot graphic assessing the risk to be free of treatment discontinuation due to neurologic adverse events at rimonabant dosages of 5 and 20 mg**.

By performing a sub-analysis of the psychiatric adverse events forcing treatment discontinuation we observed that only rimonabant at dosage of 20 mg was associated with an increased risk of psychiatric disorders (RR 2.35; 95%CI 1.66-3.34), a difference which remains after pooling both dosages (RR 1.79; 95%CI 1.24-2.58) (Figure 4). Similarly, 20 mg rimonabant and pooled data of the two dosages were associated with increased risk for treatment discontinuation due nervous system adverse events (RR 2.35; 95%CI 1.49-3.70 and, RR 1.89; 95%CI 1.30-2.75, respectively) (Figure 5).

The analysis of the number needed to harm (NNH) demonstrates a reduced NNH for the occurrence of any adverse event (NNH 22, for rimonabant 20 mg), and discontinuation due to adverse events (NNH 18, for rimonabant 20 mg). It was also found that for every 30 patients treated with 20 mg rimonabant, one will discontinue the treatment due to psychiatric adverse events (Table 2).

**Table 2:** Number need to harm (NNH) to each adverse event assessed in the meta-analysis.

	Rimonabant treated	Event	Placebo treated	Event	CER (%)	EER (%)	NNH
Any adverse event							
Rimonabant 5 mg	2883	2364	1770	1434	81	82	102
Rimonabant 20 mg	3393	2861	2292	1829	80	84	22
Total	6276	5225	4062	3263	80	83	34
Serious adverse events							
Rimonabant 5 mg	2883	149	1770	79	4	5	142
Rimonabant 20 mg	3393	207	2292	123	5	6	136
Total	6276	356	4062	202	5	6	143
Discontinuation due to adverse events							
Rimonabant 5 mg	2883	232	1770	125	7	8	102
Rimonabant 20 mg	3815	501	2708	206	8	13	18
Total	6698	733	4478	331	7	11	28
Discontinuation due to psychiatric adverse events							
Rimonabant 5 mg	2883	81	1770	42	2	3	229
Rimonabant 20 mg	3815	230	2708	72	3	6	30
Total	6698	311	4478	114	3	5	48
Discontinuation due to neurologic adverse events							
Rimonabant 5 mg	2883	26	1770	13	1	1	597
Rimonabant 20 mg	3684	78	2581	25	1	2	87
Total	6567	104	4351	38	1	2	141

CER, control event rate; EER, experimental event rate.

## Discussion

In this study we performed a meta-analysis on the adverse events related with the treatment with rimonabant, a cannabinoid-1 receptor blocker, used primarily in obesity and related disorders. We observed that the use of rimonabant at the dose of 20 mg per day is associated with adverse events including discontinuation due to psychiatric and neurologic adverse events. These results are similar to those reported in previous meta-analysis on the Rimonabant in Obesity (RIO) trials [33]. However our study included more studies as a trial for alcohol dependence which enrolled subjects without obesity but with greater risk to present adverse events (particularly psychiatric adverse events).

The NNH to discontinuation due to adverse in our study were similar to other meta-analysis (NNH 14). Of notice that this NNH is the lowest as compared to other drugs for obesity treatment (NNH 39, for orlistat; and NNH 500, for sibutramine). Given the large number of patients eligible for the treatment with rimonabant, the NNH is a concern [33].

The results of this meta-analysis also suggest that subjects exposed to 20 mg rimonabant may have some clinically relevant adverse events, and point to the need that both patients and investigators should be alerted about the early detection of these negative events. Since there is no evidence on the clinical utility and due to the proved evidence of increased adverse events rate, rimonabant cannot be recommended as a treatment option in NAFLD. This is further supported by the finding that despite possible reduction in the hepatic fat content [13], no evidence regarding histological improvement exists also because of the premature stop of rimonabant registered trials in NALFD [14,15], and other diseases (ClinicalTrials.gov registry: NCT00547118, NCT00678483, NCT00754689, NCT00434096, NCT00412698, NCT00449605, NCT00263042, NCT00690456, NCT00228176, NCT00478972, NCT00478595, NCT00405808, NCT00458081, NCT00325650, NCT00408148).

Nowadays solid information shows that rather than a totally benign disease, NAFLD may be a cause of chronic liver disease with a potential risk to develop end-stage liver disease complications that had a deleterious effect in mortality rates [34]. Considering the short period of time since its first description, all therapeutic approaches (pharmacologic or non-pharmacologic, such as dietary) had not been properly assessed, and the standard of care of this disease usually follows the guidelines of obesity-related disorders [35]. During the last 25 years the scientists demonstrate an increased interest in obesity-related liver complications, and exciting findings about the noxious relationship among the liver and fat tissue were described [34]. Promote new therapies is one face of the coin but proper detection of the potential adverse effects and/or undesirable outcomes must be carefully considered to be sure to satisfy our goal.

## Conclusion

In conclusion the use of rimonabant at 20 mg per day is associated with clinical adverse events. At present no indication on the use of this drug on NAFLD exists and therefore it cannot be suggested.

## Abbreviations

NAFLD: non-alcoholic fatty liver disease; RR: relative risk; NNH: number needed to harm.

## Competing interests

The authors declare that they have no competing interests.

## Authors' contributions

NCT conceived, design, co-ordinated, collect, analyzed, interpreted data, wrote and approved the manuscript. FTA collected, analyzed, interpreted data, corrected and approved the manuscript. GB analyzed, interpreted data, corrected and approved the manuscript. LC provided general advice, read and approved the manuscript. FM provided general advice, read and approved the manuscript. CT interpreted data, corrected and approve the manuscript.

## Pre-publication history

The pre-publication history for this paper can be accessed here:

http://www.biomedcentral.com/1471-230X/9/75/prepub

## Supplementary Material

Additional file 1 **Figure S1**. Funnel plot graphic, indicating bias in those studies reporting: a) serious adverse events, b) discontinuation due to adverse effect, and c) discontinuation due to psychiatric disorders. Click here for file

Additional file 2 **Figure S2**. Forest plot graphic assessing the risk to be free of present serious adverse events at rimonabant dosages of 5 and 20 mg. Click here for file
