# Supplementary figures and images for: Systematic review and meta-analysis on the adverse events of rimonabant treatment: Considerations for its potential use in hepatology
Source: BMC Gastroenterol. 2009 Oct 9;9:75. doi: 10.1186/1471-230X-9-75 (PMC2763866; doi:10.1186/1471-230X-9-75)

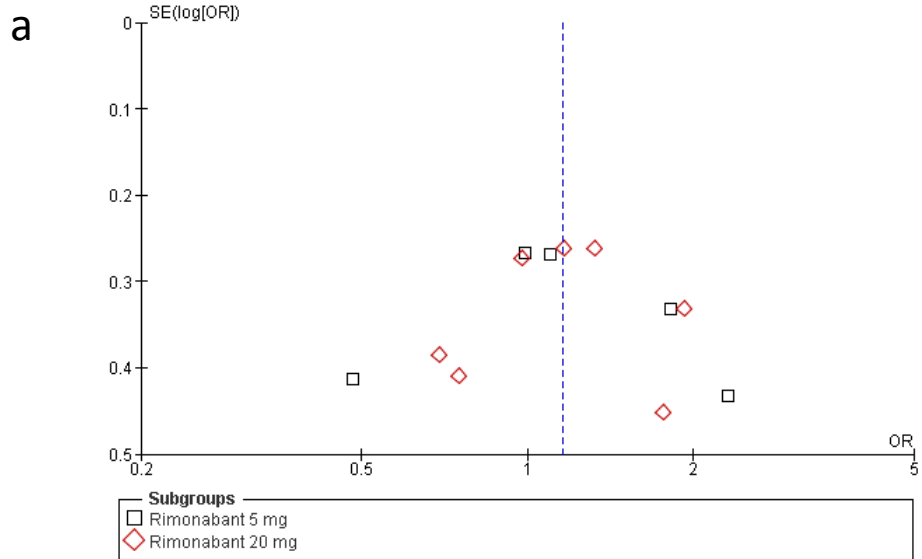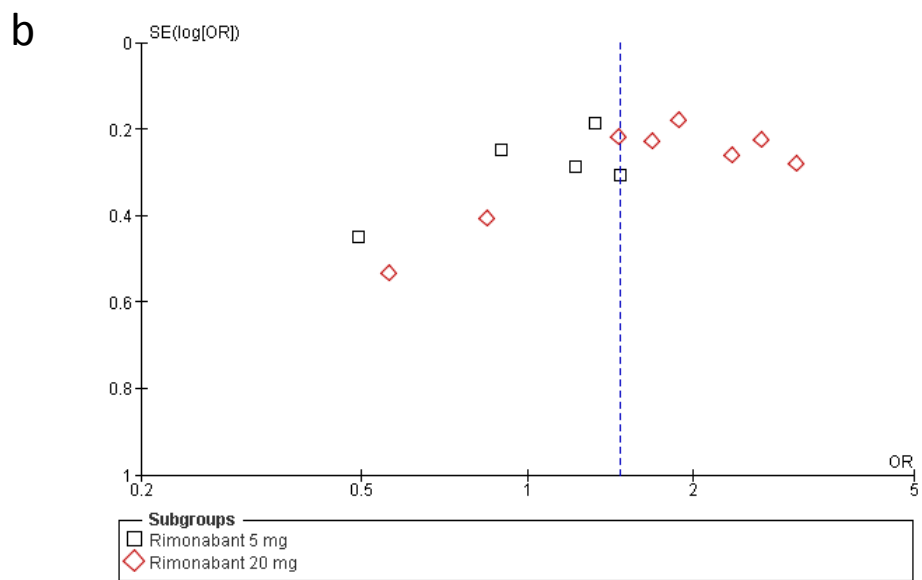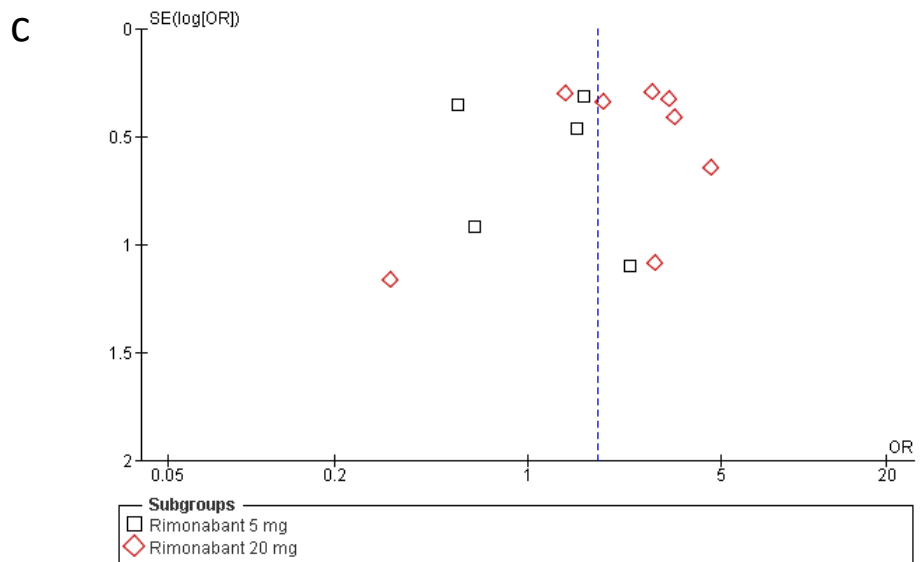

Supplement: Additional file 1 — Figure S1. Funnel plot graphic, indicating bias in those studies reporting: a) serious adverse events, b) discontinuation due to adverse effect, and c) discontinuation due to psychiatric disorders. [file 1471-230X-9-75-S1.PDF]

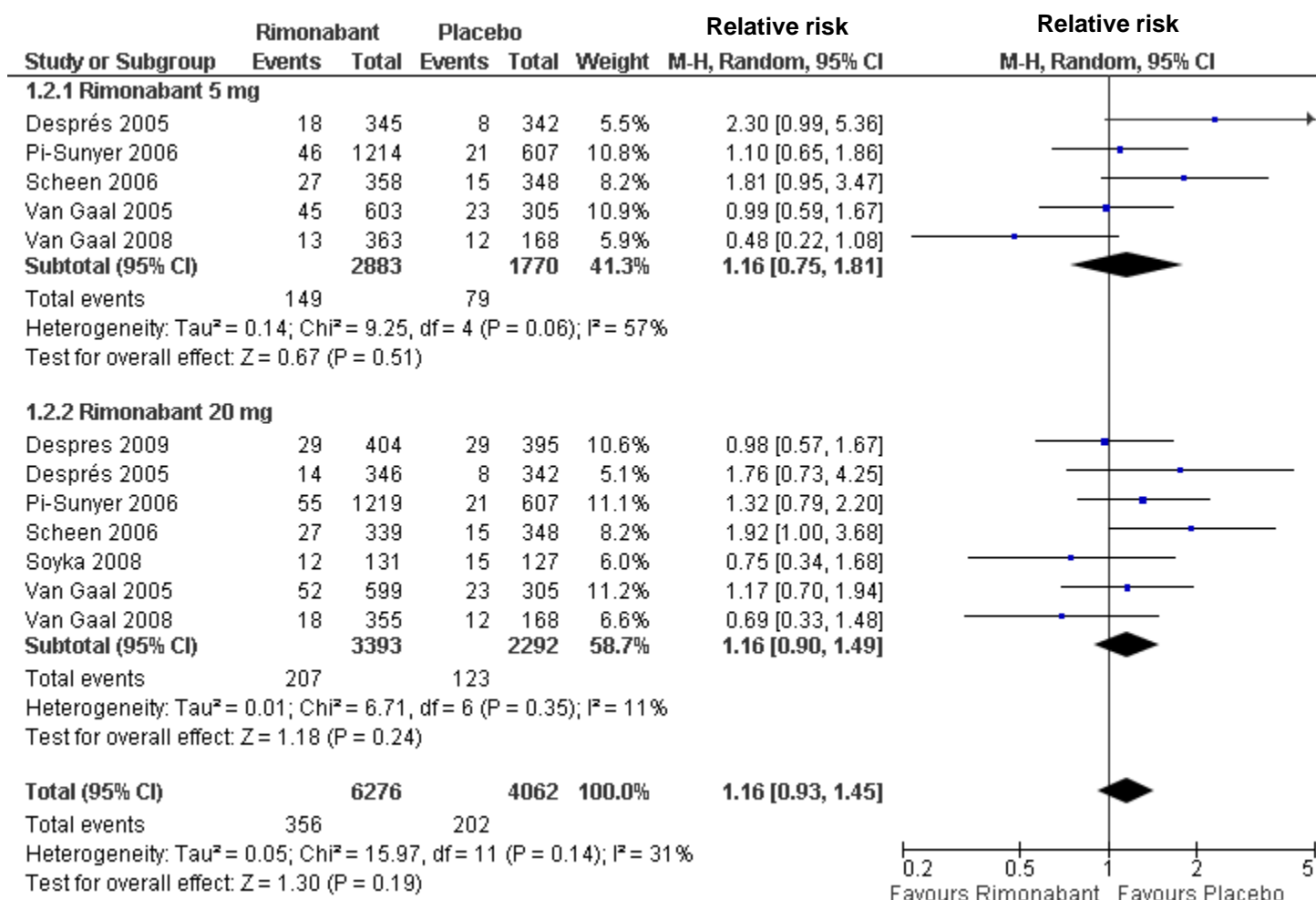

Supplement: Additional file 2 — Figure S2. Forest plot graphic assessing the risk to be free of present serious adverse events at rimonabant dosages of 5 and 20 mg. [file 1471-230X-9-75-S2.PDF]
